# Access, demand, and utilization of childhood immunization services: A cross-sectional household survey in Western Area Urban district, Sierra Leone, 2019

**DOI:** 10.7189/jogh.10.010420

**Published:** 2020-06

**Authors:** Leora R Feldstein, Roberta Sutton, Mohamed F Jalloh, Lauren Parmley, Maria Lahuerta, Adewale Akinjeji, Anthony Mansaray, Oliver Eleeza, Tom Sesay, Shibani Kulkarni, Laura Conklin, Aaron S Wallace

**Affiliations:** 1 Global Immunization Division (GID), Centers for Disease Control and Prevention, Atlanta, Georgia, USA; 2 Epidemic Intelligence Service, Centers for Disease Control and Prevention, Atlanta, Georgia, USA; 3 ICAP at Columbia University, Mailman School of Public Health, New York, New York, USA; 4 Department of Epidemiology, Mailman School of Public Health, New York, New York, USA; 5 Child Health/EPI Program, Sierra Leone Ministry of Health and Sanitation, Freetown, Sierra Leone; 6 Evaluation Fellow (Oak Ridge Institute for Science and Education), GID, Centers for Disease Control and Prevention, Atlanta, Georgia, USA

## Abstract

**Background:**

Urban childhood immunization programs face unique challenges in access, utilization, and demand due to frequent population movement between and within localities, sprawling informal settlements, and population heterogeneity. We conducted a cross-sectional household survey in the Western Area Urban district, Sierra Leone, stratified by slums and non-slums as defined by the United Nations Development Program.

**Methods:**

Based on data from child vaccination cards, weighted vaccination coverage was estimated from 450 children aged 12-36 months (household response rate = 83%). Interviews with 444 caregivers identified factors related to accessing routine immunization services. Factors associated with coverage in bivariate analyses were examined in multivariate models using backward stepwise procedure.

**Results:**

Coverage was similar in slums and non-slums for 3-doses of diphtheria-tetanus-pertussis-hepatitis B-*Haemophilus influenzae* type b (pentavalent) vaccine (86%, 92%) and second dose of measles vaccine (33%, 29%). In a multivariate logistic regression model, incomplete pentavalent vaccine coverage was associated with being second or later birth order (adjusted odds ratio (aOR) = 4.5 (95% confidence interval (CI) = 1.4-14.9), a household member not approving of childhood vaccinations (aOR = 7.55, 95% CI = 1.52-37.38), self-reported delay of child receiving recommended vaccinations (aOR = 4.8, 95% CI = 1.0-22.1), and living in a household made of natural or rudimentary materials (aOR = 3.5, 95% CI = 1.2-10.6). Overall, the majority (>70%) of caregivers reported occupation as petty trader and <50% reported receiving vaccination information via preferred communication sources.

**Conclusions:**

Although vaccination coverage in slums was similar to non-slums, study findings support the need for targeted interventions to improve coverage, especially for the second dose of measles vaccine to avoid large scale measles outbreaks. Strategies should focus on educating household members via preferred communication channels regarding the importance of receiving childhood vaccinations on time for all offspring, not just the first born. Vaccination coverage could be further improved by increasing accessibility through innovative strategies such as increasing the number of vaccination days and modifying hours.

The Expanded Program on Immunization (EPI) was established by WHO in 1974 to ensure all children had access to routinely recommended vaccines that prevent morbidity and mortality from six diseases [1,2]: tuberculosis, polio, measles, diphtheria, tetanus, and pertussis. Since 2008, there has been a global reduction in deaths due to vaccine-preventable diseases (VPDs) among children under the age of five. Nevertheless, according to the World Health Organization (WHO), in 2008 alone, there were more than 1.5 million deaths among this age group due to VPDs, 46% of whom lived in Africa [3]. Despite efforts to make vaccines available and accessible to all eligible children around the world, almost 20 million children were under- or unvaccinated in 2018 [3,4].

Sierra Leone established the national EPI in 1978 and the program now includes vaccines against five additional diseases: yellow fever, rotavirus, hepatitis B, *Haemophilus influenzae* type b, and pneumococcal disease [5]. From 1986–1990, national coverage of routine vaccinations (from WHO and UNICEF), including the third dose of diphtheria-tetanus-pertussis-containing vaccine (DTPcv), increased from 6% to 75% in Sierra Leone during the final phase of the Universal Child Immunization initiative [6]. Vaccine coverage declined, however, during the 1991-2002 country’s civil conflict and the 2014-2015 Ebola Virus outbreak [7]. As of 2018, national routine coverage with three doses of DTPcv and the first dose of measles-containing vaccine (MCV1) were estimated to be 90% and 80%, respectively [7]. Along with numerous health systems-related setbacks caused by the Ebola outbreak, suboptimal routine vaccination coverage remains a major public health concern in Sierra Leone. Despite reported improvements in coverage following the Ebola outbreak, the country has experienced multiple measles outbreaks, including one as recently as June 2018 [8]. To prevent measles outbreaks by achieving herd immunity within the population, it is estimated that coverage of at least 95% for two doses of MCV needs to be sustained [4].

Almost 15% of Sierra Leone’s population resides in Western Area Urban (WAU) district where the country’s capital Freetown is located [9]. Compared to rural areas, urban areas like Freetown may be assumed to have higher uptake of essential health care services including immunization, given the greater geographic accessibility to health facilities and availability of diverse information sources and social networks. However, the opposite is often true [10]. Vaccination coverage in WAU from the Multiple Indicator Cluster Survey (MICS) (86% for the third dose of DTPcv and 81% for MCV1), was similar to the 2018 national coverage [11]. Urban immunization programs face unique challenges, such as frequent population movement between and within localities, sprawling of informal settlements, and heterogeneity of the population [12]. Informal settlements and slum communities within urban areas have populations with generally low socioeconomic status which may result in gaps and inequities in the uptake of health services, particularly for childhood immunizations [13]. To the best of our knowledge, no data were previously available to determine if coverage varied by slum and non-slum areas of WAU district, Sierra Leone as of 2017. Slums were defined by the United Nations Development Program as having: lack of basic services, sub-standard housing or illegal and inadequate building structures, overcrowding and high density, unhealthy living conditions and hazardous locations, insecure tenure, irregular and informal settlements [14].

Our primary research question of interest was to understand the relationship between a child’s residence (slum and non-slum) and their vaccination status. We conducted a cross-sectional household survey in WAU district, stratified by designated slum and non-slums to estimate vaccination coverage among children 12-36 months old and identify factors contributing to underutilization of, and barriers to accessing routine immunization services. The objective was to provide actionable data to guide the Sierra Leone Ministry of Health and Sanitation (MoHS) to improve access, demand, and utilization of immunization services by tailoring and strengthening immunization services in Freetown and other similar urban settings in the country.

## METHODS

### Study setting and population

In WAU district, routine childhood vaccinations are administered by trained health care workers at fixed sites within health facilities, as well as through outreach visits, and designated routine immunization days in a central community location. The current childhood vaccination schedule in Sierra Leone follows the recommended WHO vaccination schedule. It includes a birth dose of bacillus Calmette-Guérin (BCG) and oral polio vaccine (OPV); doses of OPV, diphtheria-tetanus-pertussis-hepatitis B-*Haemophilus influenzae* type b (pentavalent) vaccine, pneumococcal, and rotavirus vaccine at 6 weeks, 10 weeks, and 14 weeks of age; yellow fever vaccine and MCV1 at 9 months; and MCV2 at 15 months of age. Pneumococcal vaccine, rotavirus vaccine, and MCV2 were all introduced into the national schedule within the past eight years (2011, 2012, and 2015, respectively) [5]. As a result of MCV2 introduction, the Sierra Leone MoHS and global partners have placed a renewed emphasis on strengthening child health interventions during the second year of life.

### Study design

A household survey was conducted with eligible caregivers of children aged 12-36 months in WAU district, Sierra Leone from March 4 to April 8, 2019. Two-stage probability sampling methodology was used to randomly select 58 enumeration areas (EA) from the 2015 Sierra Leone Census, stratified by setting (29 slum EAs and 29 non-slum EAs according to the designated definition), and simple random sampling was used to select 10 eligible households (physical structures or dwellings) per EA from a newly created EA household line listing (Appendix S1 in the **Online Supplementary Document**). All eligible children within a household were selected for inclusion in the study, and their primary caregivers were selected for interviews. The data collection teams visited the selected EAs at varying times of the day, including mornings, afternoons, and evenings to increase the chances of locating eligible participants on the enumerated list. If a caregiver was not present, data collectors returned to the household up to three times at another time of the day or on a different day. Households with unreachable caregivers were not replaced or substituted. Individuals were eligible to participate if they were aged 15 years and older, living in residential households in the selected EAs for the past 6 months, and were primary caregivers of children aged 12-36 months.

### Data collection

Caregivers of children aged 12-36 months were interviewed near their households, using a tablet-based respondent questionnaire. Key topics included knowledge of available immunization services, access to information and services, barriers and facilitators to vaccination-seeking behavior including attitudes toward vaccines, and influencers at household, community and societal levels. Data from the child’s vaccination card was abstracted and entered into tablets to ascertain the immunization status of the child. Caregiver recall was also collected those children without vaccination cards.

### Ethics approval and consent

The survey was approved by Columbia University Medical Center’s Institutional review board, the Centers for Disease Control and Prevention (CDC) Office of the Associate Director for Science, Center for Global Health, and Sierra Leone’s MoHS institutional review board. Written or thumb-printed (for illiterate participants) informed consent was obtained from participants in either English or Krio. If the caregiver was <18 years, the parent of the caregiver was asked to consent, and minor assent was obtained. Caregivers were excluded from the survey if they were unable or unwilling to provide consent. Participation was voluntary, and the objectives, length of survey, and risks/benefits of participation were explained to individuals prior to enrollment.

### Statistical analysis

Descriptive statistics were used to summarize demographic data, as well as access, demand, and utilization of childhood vaccinations in slum and non-slums of WAU. From collected vaccination card data, estimations of vaccination coverage and the corresponding 95% confidence intervals of point estimates were made using complex survey procedures that accounted for the two-stage cluster design and sampling weights [15]. A timeliness analysis to assess whether children had received all of their vaccinations within 24 months of age was conducted among all children who were age-eligible (15 months or older) for MCV2 – the final vaccine dose in the national childhood immunization schedule. χ^2^ tests were used to examine differences between slum and non-slums in uptake of various scheduled vaccines, access-related determinants, and attitudes toward vaccination. A multivariable logistic regression model was fit to the data to examine the odds of incomplete pentavalent vaccination status among children of caregivers who resided in slum communities compared to those residing in non-slum communities. Variables considered for inclusion in the model were significantly associated (*P* < 0.05) with either geographic location (slum vs non-slum) or complete vaccination coverage of pentavalent vaccine in the bivariate analysis. When using a manual backward stepwise procedure, these variables were retained in the multivariate analysis if they remained significant at the 0.05 level. All analyses were completed in STATA 14 (StataCorp, College Station TX, USA).

## RESULTS

### Enrollment

**Figure 1:**
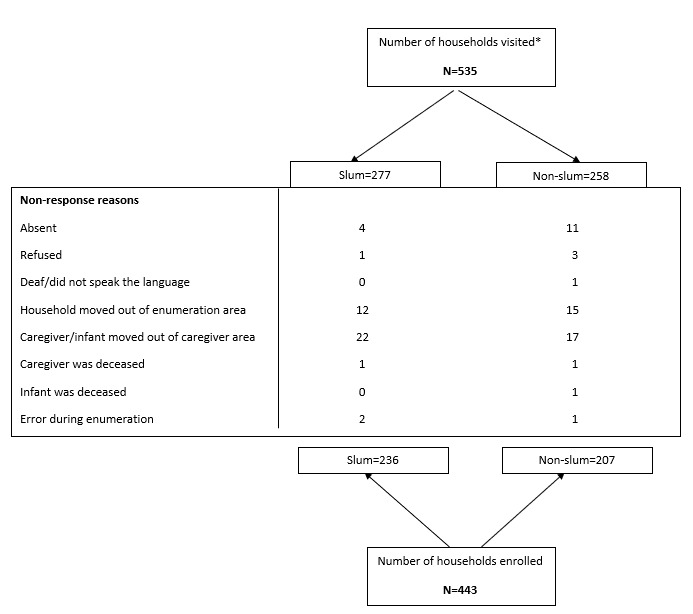
Households included in the analysis and reasons for exclusion, (unweighted), household survey, WAU district, Sierra Leone, 2019. *Visited households are defined as all eligible households (physical structures or dwellings) that were visited by data collection teams up to three times. WAU – Western Area Urban

A total of 4275 structures comprising 7869 households were enumerated in the 58 randomly selected EAs, of which 1268 households were determined to be eligible for inclusion in the study. At least 80% of households in each EA provided household listing information. Data collection teams visited 535 households selected from a created list of eligible households; 277 (52%) were in slums and 258 (48%) were in non-slums (**Figure 1**). Questionnaires were completed from 443 households with an overall response rate of 83%. Eligible caregivers, infants, or entire households who had moved out of the area since enumeration (72%) comprised the majority of the non-responders. Of the 443 completed households, 454 caregivers of 470 children aged 12-36 months were interviewed. There were 10 children and their caregivers excluded from analyses due to being age-ineligible (**Table 1**). Therefore, the final analysis included data from 444 caregivers and 460 children (240 children from 236 households in slums and 220 children from 207 households in non-slums).

**Table 1:** Caregivers and eligible children included in the analysis by slum and non-slum areas, (unweighted), household survey, Western Area Urban district, Sierra Leone, 2019

	Slum, n (%)	Non-slum, n (%)	Total, N
Number of caregivers interviewed	240 (53)	214 (47)	454
First enrolled caregiver within household	236 (53)	207 (47)	443
Second enrolled caregiver within household	4 (36)	7 (66)	11
Number of caregivers included in analysis*	234 (53)	210 (47)	444
Number of participating children	246 (52)	224 (48)	470
First enrolled child within household	236 (53)	207 (47)	443
Second enrolled child within household	6 (38)	10 (62)	16
Third enrolled child within household	4 (36)	7 (66)	11
Number of eligible children enrolled*	240 (52)	220 (48)	460

WAU – Western Area Urban

*10 children and caregivers were enrolled and interviewed but excluded during the analysis phase because the children were not between 12-36 months of age either at the time of enumeration or at the time of interview.

### Demographic characteristics of children and caregivers

The median age of participating children was 24 months in slums and 25 months in non-slums (**Table 2**). Forty-six percent (n = 109) and 53% (n = 117) of these children were female in slum and non-slums, respectively. The median birth order of participating children was second for both slum and non-slums, and the majority of participating caregivers were mothers (90% and 89%, respectively). The median maternal age was 26 years in both areas (**Table 2**). The majority of mothers in slum and non-slums were married (67% (n = 156) and 64% (n = 134), respectively), Muslim (78% (n = 182) and 62% (n = 119), respectively), and considered themselves to be petty traders (67% (n = 156) and 54% (n = 113), respectively). A smaller proportion of mothers in slums reported completing junior secondary school or higher compared to mothers in non-slums (45% and 60%, respectively).

**Table 2:** Child, caregiver, and household characteristics comparing slum to non-slum areas (unweighted), household survey, Western Area Urban district, Sierra Leone, 2019

	Slum (n = 240)		Non-slum (n = 220)		
**Child characteristics**	**n or (median)**	**(Range) or %**		**n or (median)**	**(Range) or %**
Median age of child in months*	(24)	(13-41)		(25)	(12-39]
Median birth order	(2)	(1-10)		(2)	(1-8]
First birth order	63	47%		71	53%
Sex (female)	109	46		117	53
	**Slum (n = 234)**			**Non-slum (n = 210)**	
**Caregiver/parent characteristics**	**n or (median)**	**(Range) or %**		**n or (median)**	**(Range) or %**
**Caregiver (respondent) type:**					
Mother	210	90		186	89
Father	4	2		0	0
Grandmother	14	6		17	8
Grandfather	0	0		1	<1
Other family member†	5	2		6	3
**Median maternal age**	(26)	(18-46)		(26)	(17-48]
**Mother no longer alive**	0	0		1	<1
**Maternal marital status:**					
Married	156	67		134	64
Living with partner	39	17		24	11
Divorced/separated or widowed	7	3		6	2
Never married/never lived with partner	31	13		45	22
**Maternal education status:**					
Mother never attended school	73	32		35	17
Mother attended some or completed primary school	55	24		83	23
Mother completed junior secondary school or higher	103	45		126	60
**Paternal education status:**					
Father never attended school	44	21		32	16
Father attended some or completed primary school	27	13		16	8
Father completed junior secondary school or higher	136	66		146	75
**Maternal religion:**					
Christian	50	21		80	38
Muslim	182	78		119	62
Paternal religion:					
Christian	53	23		58	28
Muslim	176	77		150	72
**Maternal employment:**					
Petty trader	156	67		113	54
Private business owner	14	6		25	12
Unemployed	27	12		43	21
Student	8	3		12	6
Medical or health professional	2	1		5	2
Other (laborer, teacher, government or NGO employee)‡	26	12		11	5
**Paternal employment:**					
Petty trader	29	13		20	10
Laborer	74	32		54	26
Private business owner	40	17		37	18
Government employee	18	8		26	12
Public transportation or Okada/Keke driver	22	10		19	9
Unemployed	5	2		12	6
NGO employee	5	2		9	4
Teacher	3	1		7	3
Other (farmer, medical professional, solider, student)‡	33	14		25	12
	**Slum (n = 236)**			**Non-slum (n = 207)**	
**Household level characteristics**	**n or (median)**	**(Range) or %**		**n or (median)**	**(Range) or %**
Median household size	(5)	(2-23)		(5)	(1-20]
Median number of living children in household	(2)	(1-8)		(2)	(1-6]

NGO – non-governmental organization

*Age at time of enumeration, calculated from date of birth on the vaccination card. If vaccination card was unavailable, it was calculated from date of birth based on caregiver recall.

†Other family members include: aunt, cousin, niece, nephew, or sibling.

‡Categories with ≤2% were grouped into “Other”.

### Household characteristics

Although household size ranged from 1-23 individuals, median size was five in both slum and non-slums (**Table 2**). Most households (82% (n = 195) in slums and 74% (n = 153) in non-slums) had access to piped or dug well water but did not have these potable water sources in their homes or on their land (72% (n = 143] in slums and 74% (n = 122) in non-slums) (Table S1 in the **Online Supplementary Document**). Almost a quarter (24%, n = 56) of homes in slums used a bucket, hanging toilet or plastic bag as toilet facilities, compared to 5% (n = 10) of households in non-slums. A greater proportion of households in non-slums had electricity, a radio, television, mobile phone, and refrigerator than households in slums; 43% (n = 88) and 29% (n = 69), respectively. A greater proportion of non-slums also had rudimentary or finished walls, floors, and roof compared to households in slums (94% (n = 207) and 89% (n = 213), respectively).

### Access to and utilization of routine immunization

Caregiver access and utilization of routine immunization did not differ significantly between slum and non-slums. Caregivers in both areas reported similar travel time from their home to vaccination sites and similar wait times at vaccination sites (Table S2 in the **Online Supplementary Document**). More than one quarter (34%-40%) of all caregivers reported that transportation time to vaccination sites was “too much time”. When asked about preferred location for an additional vaccination site, the majority of caregivers reported that they would like a site close to home (88%-90%) compared to close to work or the market. In both areas, 79% of caregivers reported that wait time at vaccination sites was ≥30 minutes and 14%-22% of caregivers viewed this as “too much time”. Half of all caregivers (50%) reported having to pay health care workers for routine immunization services, even though services are supposed to be free of charge. One in ten caregivers reported attending a vaccination site but having to return home without their child being vaccinated. Among caregivers returning home without getting their children vaccinated, the three main reasons reported for missed opportunities for vaccination were: “not enough children to open a vaccination vial”, “vaccines were not available”, and “it was not a scheduled vaccination day”.

Very few caregivers self-reported refusing recommended vaccinations for their child (2%-6%); however, 36%-37% of caregivers self-reported delaying vaccination for their child (Table S2 in the **Online Supplementary Document**). Of the 17 caregivers who self-reported vaccine refusal, 10 cited fear of vaccination side effects (Table S3 in the **Online Supplementary Document**). Of the 163 caregivers who self-reported vaccine delay, the top three reasons were: lack of time to take child, child or caregiver were sick, and fear of vaccination side effects.

### Current and preferred sources of information about vaccines

Sixty-two percent of caregivers in slums and 75% of caregivers in non-slums received information about childhood vaccinations from health facilities. However, only 43% of caregivers in slums and 47% of caregivers in non-slums reported that health facilities were their preferred source of information (data not shown). Caregivers in both areas reported that household visits, radio programming, and community-based events were their preferred modes of communication relating to childhood vaccination.

### Perceptions of childhood vaccination

Perceptions of childhood vaccination were similar among caregivers in slum and non-slums. A high proportion of caregivers (≥90%) in both slum and non-slums reported that they believe vaccines are good for their children, are safe, and protect their children against diseases (Table S4 in the **Online Supplementary Document**). When caregivers were asked whether they would encourage others to get their children vaccinated, 67% in slums and 73% in non-slums responded, “very much.” Less than 80% of caregivers in both areas reported that parents and trusted leaders in the community “very much” approved and valued childhood vaccinations.

### Vaccination coverage

Vaccination card retention at the time of interview was similar in slum and non-slums (75% and 76%, respectively) (**Table 3**). Using vaccination card data only, BCG vaccination coverage was high in both areas (≥98%). Overall, pentavalent vaccination coverage was similar between slum and non-slums; 97% for the first dose, 91% for two doses, and 86% for all three doses in slums and 96% for the first dose, 94% for two doses, and 92% for all three doses in non-slums. Similar to pentavalent vaccine coverage, there was an observed drop in coverage between the first and second measles doses; coverage was similar in slums and non-slums, with coverage for MCV2 (32%-33%) lower than coverage for MCV1 (75%-77%).

**Table 3:** Vaccination coverage of children ages 12-36 mo and characteristics comparing slum to non-slum areas (weighted), household survey, Western Area Urban district, Sierra Leone, 2019*

Vaccination coverage	Slum (n = 238†)		Non-slum (n = 220)		Total (n = 458)	
	**% or (mean)**	**95% CI**	**% or (mean)**	**95% CI**	**% or (mean)**	**95% CI**
**Mean age of most recent vaccination in months (recall)**	(11)	11-12	(11)	11-12	(11)	11-12
**Owns vaccination card:**						
Yes, available	75	69-81	76	70-80	76	71-80
Yes, not seen	19	15-25	17	13-22	17	14-22
No	6	3-10	7	3-15	7	4-14
**Vaccination coverage by card**	**n = 180**		**n = 170**		**n = 350**	
BCG vaccination	98	94-99	100	98-100	99	98-100
Penta1	97	94-99	96	83-100	96	86-99
Received two doses of penta	91	85-95	94	84-98	94	86-97
Received all three penta doses	86	80-91	92	83-97	91	84-96
MCV1	75	66-83	79	66-86	77	68-85
MCV2‡	33	25-42	32	21-46	32	22-44
Received BCG, all penta doses and 2 measles doses‡	33	25-42	29	19-42	29	20-40
Received BCG, all penta doses and 2 measles doses by 24 months§	22	16-30	20	14-29	20	15-28
No vaccinations	1	1-5	0	-	<1	<1-1
Received all recommended vaccinations‡ (recall)	54	43-63	53	41-64	53	43-62

CI – confidence interval, BCG - Bacillus Calmette-Guérin; Penta - pentavalent vaccine (diphtheria-tetanus-pertussis-hepatitis B-*Haemophilus influenzae* type b), MCV1 – measles-containing vaccine, 1^st^ dose; MCV2 – measles-containing vaccine, 2^nd^ dose

*No significant differences at *P* < 0.05 level

†Missing data from two participants.

‡All recommended vaccines include BCG, Penta1-3, MCV1-2, and 4 doses of oral polio vaccine.

§Only children ≥15 months were included in the analysis.

According to caregiver recall, 54% of children in slums and 53% of children in non-slums received all recommended vaccines in the national routine schedule; however, according to vaccination card data, only 33% of children in slums and 29% of children in non-slums received BCG, three doses of pentavalent, and two doses of measles vaccine. Of the caregivers who reported that their child received all recommended vaccines, only 8% (95% CI = 3%-18%) had vaccination cards with documented receipt of MCV2. In comparison, 60% (95% CI = 44%-74%) received MCV1, and 86% (95% CI = 74%-93%) received the third dose of pentavalent vaccine (data not shown). Less than one quarter of all age-eligible children received these doses on time (by 24 months of age) (**Table 3**). Among children with vaccination cards (n = 350), 1% of children in slums and 0% of children in non-slums had never been vaccinated. Similar trends in vaccination coverage, by card and recall, among the 12 to 23 month-old birth cohort were observed (Table S5 in the **Online Supplementary Document**).

### Factors associated with vaccination coverage

In bivariate analyses, failure to complete the pentavalent vaccination series (from vaccination card only) was significantly associated with second or higher birth order of the child, paternal education of primary school or less, living in a house without a flush or pour toilet, and living in a house with natural floor, roof, and wall materials (**Table 4**). Failure to complete the pentavalent vaccination series was significantly higher among children with caregivers who knew a child in their family or community with vaccine side effects in the last 12 months and those who self-reported delaying recommended vaccinations for their children (**Table 5**). Children who had not received three doses of pentavalent vaccine had caregivers who less frequently perceived that their spouses and household members “very much” approved of childhood vaccination and had caregivers who less frequently reported being confident in their ability to take their child for vaccination visits, (*P* < 0.01 and *P* = 0.02, respectively) (S5 Table). Children were also less likely to have received three doses of pentavalent vaccine series if their caregivers less frequently perceived that they lived in a community where people valued childhood vaccination and approved of childhood vaccination (*P* = 0.03 and *P* = 0.01, respectively).

**Table 4:** Demographic and socioeconomic factors associated with receiving three doses of pentavalent vaccine among children who had a vaccination card at the time of interview* (weighted), household survey, WAU district, Sierra Leone, 2019

	All three pentavalent vaccine doses		*P*-value
	**%**	**95% CI**	
**Urban setting:**			
Slum	86	80-91	0.18
Non-slum	92	83-97	
**Gender of child:**			
Female	94	83-98	0.24
Male	88	79-94	
**Maternal education level:**			
Never attended school	89	73-96	0.28
Attended some or completed primary school	87	72-94	
Completed junior secondary school or higher	94	83-98	
**Paternal education level**			
Never attended school	79	58-91	0.03
Attended some or completed primary school	95	87-98	
Completed junior secondary school or higher	94	85-98	
**Maternal religion:**			
Christian	95	86-98	0.34
Muslim	90	79-95	
**Paternal religion:**			
Christian	95	84-99	0.33
Muslim	90	81-95	
**Child birth order:**			
1^st^ born	98	95-99	<0.001
≥2^nd^ born	88	77-94	
Maternal marital status:			
Married or living together	90	80-95	0.02
Divorced, separated or widowed	94	61-99	
Never married or lived together	98	93-100	
Type of toilet:			
Flush or pour flush toilet	98	95-99	0.002
Pit Latrine, bucket, hanging toilet or plastic bag	88	77-94	
**Household amenities composite variable (electricity, mobile phone, radio, television and refrigerator):**			
Has household amenities	92	77-97	0.93
Does not have household amenities	91	85-95	
**Main material of house (floor, roof and walls):**			
Natural materials	68	28-92	0.01
Rudimentary or finished materials	94	91-96	

WAU – Western Area Urban, CI – confidence interval

*n = 350 children 12-36 months with vaccination card.

**Table 5:** Caregiver characteristics, perceptions, and decisions associated with receiving three doses of pentavalent vaccine among children who had a vaccination card at the time of interview* (weighted), household survey, WAU district, Sierra Leone, 2019

	All three pentavalent vaccine doses	
	**%**	**95% CI**
**Transportation time from home to vaccination site:**		
<30 min	92	83-97
30 min-1 h	92	82-97
>1 h	93	68-99
**Perception of time to reach usual vaccination site**		
About right	94	86-98
A short time	91	83-95
Too much time	93	77-98
**Waiting time at vaccination site:**		
<30 min	82	57-94
30 min-1 h	96	91-98
>1 h	94	85-98
**Perception of wait time at vaccination site:**		
About right	95	90-98
A short time	89	79-95
Too much time	89	67-97
**Payment to health care worker:**		
Nothing	95	87-98
1000-5000 Leones	92	85-96
≥6000 Leones	90	75-96
**Knowledge of child in family or community with vaccine side effects within last 12 mo:**		
Yes	70	37-91
No	93	87-96
**Self-reported refusal of recommended vaccination:**		
Yes	75	39-94
No	93	87-96
**Self-reported delays in receiving recommended vaccination:**		
Yes	81	63-92
No	97	92-99

WAU – Western Area Urban, CI – confidence interval

*n = 350 children 12-36 months with vaccination card.

Children who were age-eligible but had incomplete measles vaccination were more likely to have a mother and father who reported being Muslim; incomplete coverage was 66% (95% CI = 53%-78%) among children with Muslim mothers compared to 47%, 95% CI = 30%-64%) among Christian mothers and 64% (95% CI = 50%-76%) among children with Muslim fathers compared to 42%, 95% CI = 26%-60%) among Christian fathers (data not shown). Children with incomplete measles vaccination were also more likely to live in a house without amenities including electricity, a television, radio, mobile phone, refrigerator (70%, 95% CI = 52%-83%) compared to 43%, 95% CI = 33%-55% (data not shown). Additionally, children whose caregivers delayed their vaccination were more likely to have incomplete measles vaccination coverage (34%, 95% CI = 23%-47%) compared to 11% (5%-22%) (data not shown).

In a multivariate analysis, the following covariates were most closely associated with incomplete pentavalent vaccination series: being born second or later (adjusted odds ratio (aOR) = 4.5, 95% CI = 1.4-14.9), living in a household where a spouse, partner or household member does not approve of childhood vaccinations (aOR = 7.55, 95% CI = 1.52-37.38), caregiver self-reporting delay of child receiving recommended vaccinations (aOR = 4.8, 95% CI = 1.0-22.1), and living in a household made of natural or rudimentary materials (aOR = 3.5, 95% CI = 1.2-10.6) (**Table 6**).

**Table 6:** Logistic regression model examining factors associated with incomplete pentavalent vaccine coverage (n = 41) among children ages 12-36 months living in Western Area Urban district, Sierra Leone (weighted), household survey, 2019

Did not receive all three doses of pentavalent vaccine*	Odds ratio	95% confidence interval	*P*-value
Living in a slum enumeration area	1.8	0.70-4.8	0.21
Child birth order (1^st^ vs ≥2^nd^)	4.5	1.4-14.9	0.01
Caregiver perceives that household member does not approve of vaccination	8.7	2.2-34.2	0.002
Self-reported delays in receiving recommended vaccination	4.8	1.02-22.1	0.05
Main materials of household (natural vs rudimentary or finished)	3.5	1.2-10.6	0.03

*From vaccination card data only.

## DISCUSSION

Although we did not find any differences in vaccination coverage between slum and non-slums in a large urban area (WAU district) in Sierra Leone, our findings show that overall, almost 1 in 4 children had not received MCV1 and only 1 in 3 children received MCV2 vaccination. Based on the results of this survey, barriers to accessing and utilizing routine immunization services in both areas fell into four categories: 1) sociodemographic characteristics of the family, 2) caregiver perceptions of family and community beliefs, attitudes, and behaviors toward vaccination, 3) vaccination session scheduling, and 4) facility-based experiences and missed opportunities for vaccination. Of potential barriers identified, factors most closely associated with low vaccination coverage in multivariate analysis were a child being born second or later, caregivers perceived lack of support for vaccination from their spouse/partner or other household members, self-reported delay of child receiving recommended vaccinations, and living in a household made of natural or rudimentary materials.

Vaccination coverage estimates from this study for the third dose of pentavalent vaccine and MCV1 are consistent with recent estimates from other assessments in WAU district [7,11]. Findings from prior studies in sub-Saharan Africa aimed to identify predictors of vaccination coverage in urban areas and found the following: low or no maternal education level, low socioeconomic status, time constraints, past behaviors of delaying vaccination, and negative perceptions of vaccination were consistently associated with lower coverage across multiple studies [16-21]. In our study, the proportion of children who received all vaccines on time (within 24 months of age) is also low (20%) and delaying vaccination can increase the risk of child deaths due to a VPD. In comparison, two studies in urban areas of sub-Saharan Africa found a higher proportion of fully vaccinated children (55% in informal settlements in Nairobi, Kenya in 2014. and 44% in Benin City, Nigeria in 2005) [19,22].

Our finding that coverage of all routine vaccinations was similar between children in slums and non-slums was surprising. Populations within urban areas move between and within localities, and often create informal settlements, which results in heterogeneity of the population [12]. These informal settlements and slum communities within urban areas typically have gaps and inequities in the uptake of health services, particularly for childhood vaccinations. To our knowledge, this is the first time that differences in vaccination coverage have been examined between slum and non-slums within an urban setting in any African country, and although we did not identify differences in coverage, it is important to continue to assess these areas because of varying contexts and country-specific factors.

Caregivers in slums and non-slums had similar experiences, attitudes, and perceptions towards childhood vaccinations. More than one-third of all caregivers delayed vaccination for their child and thought that the time it took to reach their usual vaccination site was too long. While most caregivers believed that vaccines were safe and provided protection against diseases, approximately one quarter or more reported that parents and trusted leaders in their community were not likely to approve of childhood vaccinations and that the caregivers would not be likely to encourage other caregivers to get their child vaccinated. Further qualitative research could help shed light on how to improve confidence among caregivers to get their children vaccinated and to increase support for routine vaccination among members in the community.

To improve vaccination coverage in this urban setting of Sierra Leone, it is imperative to not only engage and educate mothers and fathers but all household members and community members in dialog, through communication channels that caregivers reported preferring (household visits, radio programming, and community-based events, in addition to health facilities). Consideration should also be given to the unique or more common media options that exist in this urban setting for sharing immunization information. Education about childhood vaccination should include why vaccines are important, how they protect against severe diseases, and risk communication about adverse events following immunization [23]. Because MCV2 coverage is so low, special attention should be brought to the importance of measles vaccination through preferred communication channels to avoid a large scale measles outbreak. Moreover, trusted local leaders (formal and informal) should be identified within diverse urban social networks and be engaged to support and role-model positive vaccination attitudes and behaviors in the community. Health facilities could consider strengthening ties with local places of worship, particularly with mosques, to reinforce vaccination messaging and provide vaccination reminders after prayer services. All communication and education messages regarding routine immunization should be developed using simple language and tested to ensure caregivers with varying degrees of education understand.

Vaccination coverage could be further improved by ensuring routine immunization services are free of charge, increasing the number of vaccination days, modifying hours, and exploring different types of outreach activities to ensure caregivers have flexibility as to when and where they bring their child for vaccination. The majority of caregivers reported that maternal occupation was “petty trader”. Thus, the market place as the most common location for petty trading should be targeted for vaccination outreach activities and demand promotion.

Educating health care workers to open vials, even if it is likely to result in some wastage could also improve coverage and enhance the relationship between the caregiver and health provider. A national policy of opening vials despite wastage and ensuring a steady supply of vaccines are needed to support this kind of health care worker education and reduce missed opportunities for vaccination [24,25]. Furthermore, more than one-third of caregivers reported delayed vaccination and delaying was associated with not getting vaccinated. In addition to encouraging health care workers to open vials when a caregiver brings a child for a vaccination session, strengthening defaulter tracking, and utilizing local community health volunteers to do so, may help to ensure that caregivers return at a later date with their child. Clear and positive communication between the provider and caregiver during vaccination sessions is also essential to ensure that the provider has fully addressed any vaccine barriers or hesitancy, such as vaccine side effects [26,27].

### Limitations

There were certain limitations to this study. EA maps, provided by Statistics Sierra Leone, lacked sufficient attributes to accurately identify boundaries and structures. Thus, some households may have been included or excluded inaccurately from an EA during listing. Steps were taken to mitigate inaccurate inclusion/exclusion during listing by collaborating with the cartography unit of Statistics Sierra Leone to conduct detailed sketch mapping of each EA before listing. The gap in time between enumeration and the household survey may have increased our non-response rate due to the mobility of households out of and between EAs during that time period. Additionally, although slum and non-slum status were defined by the United Nations Development Program, status of certain areas may have changed since the designation, resulting in misclassification. This study was not powered to examine vaccination coverage by birth cohort and thus we were unable to discern whether factors associated with low vaccination coverage differed by year and comparison to the 2017 MICS data should be done so with caution. Lastly, almost one quarter (24%) of caregivers did not have their child’s vaccination card available. This may have introduced bias because the analysis of factors associated with coverage only included children with vaccination cards.

## CONCLUSIONS

Findings from this survey highlight barriers faced by caregivers to access and utilize childhood immunization services in slum and non-slums in WAU, Sierra Leone. As a next step, the Sierra Leone MoHS and global health partners will collaborate on implementing key interventions to address identified challenges to childhood vaccination in WAU.

## Additional material

Online Supplementary Document

## References

[1] Jean Paul Uwizihiwe HB 40th anniversary of introduction of Expanded Immunization Program (EPI): A literature review of introduction of new vaccines for routine childhood immunization in sub-Saharan Africa. International Journal of Vaccines and Vaccination. 2015;1 . 10.15406/ijvv.2015.01.00004

[2] Expanded programme on immunization. Programme review. Wkly Epidemiol Rec. 1994;69:87-90. 8003403

[3] World Health Organization. Immunization, Vaccines and Biologicals: Estimates of disease burden and cost-effectiveness. Available: https://www.who.int/immunization/monitoring_surveillance/burden/estimates/en/. Accessed: 4 April 2019.

[4] World Health Organization. Progress and Challenges with Achieving Universal Immunization Coverage. Geneva, Switzerland: 2017.

[5] Sanitation GoSLMoHa. Comprehensive EPI multi-year plan: 2012-2016. 2014. Available: https://extranet.who.int/countryplanningcycles/sites/default/files/country_docs/Sierra%20Leone/cmyp-_2012-2016_narrative_for_sierra_leone_final_updated_on_the_23_jan_2014.pdf. Accessed 5 April 2019.

[6] World Health Organization. Analytical summary - Immunization and vaccines development – Sierra Leone. Available: http://www.aho.afro.who.int/profiles_information/index.php/Sierra_Leone:Analytical_summary_-_Immunization_and_vaccines_development. Accessed: 4 April 2019.

[7] World Health Organization and UNICEF. Sierra Leone: Estimates of immunization coverage: 2017 revision. Available: http://apps.who.int/immunization_monitoring/globalsummary/estimates?c=SLE. Accessed: 4 April 2019.

[8] World Health Organization. Measles outbreak confirmed in northern Sierra Leone. Available: https://afro.who.int/news/measles-outbreak-confirmed-northern-sierra-leone. Accessed: 4 April 2019.

[9] Statistics Sierra Leone. 2015 Population and Housing Census, summary of final results. Freetown, Sierra Leone: 2015.

[10] Crocker-Buque T Mindra G Duncan R Mounier-Jack S Immunization, urbanization and slums - a systematic review of factors and interventions. BMC Public Health. 2017;17:556. 10.1186/s12889-017-4473-7 28595624 PMC5465583

[11] Statistics Sierra Leone - SSL. Sierra Leone Multiple Indicator Cluster Survey, 2017, Survey Findings Report Freetown, Sierra Leone: Statistics Sierra Leone, 2018.

[12] Nelson KN Wallace AS Sodha SV Daniels D Dietz V Assessing strategies for increasing urban routine immunization coverage of childhood vaccines in low and middle-income countries: A systematic review of peer-reviewed literature. Vaccine. 2016;34:5495-503. 10.1016/j.vaccine.2016.09.038 27692772 PMC5309783

[13] UNICEF. Advantage or Paradox? The challenge for children and young people of growing up urban. 2018.

[14] The United Nations Development Programme and The Government of Sierra Leone. The Improvement of Slums and Informal Settlements in Freetown. 2006.

[15] World Health Organization. Immunization coverage cluster survey—Reference manual Available: http://apps.who.int/iris/bitstream/10665/69087/1/WHO_IVB_04.23.pdf. Accessed: 20 September 2019.

[16] Landoh DE Ouro-Kavalah F Yaya I Kahn A-L Wasswa P Lacle A Predictors of incomplete immunization coverage among one to five years old children in Togo. BMC Public Health. 2016;16:968. 10.1186/s12889-016-3625-5 27618851 PMC5020474

[17] Douba A Aka LBN Yao GHA Zengbé-Acray P Akani Bangaman C Konan NG Facteurs sociodémographiques associés à la vaccination incomplète des enfants de 12 à 59 mois dans six pays d’Afrique de l’ouest. [in French]. Sante Publique. 2015;27:723-32. 10.3917/spub.155.0723 26752038

[18] Ndiaye NM Ndiaye P Diedhiou A Gueye AS Tal-Dia A Factors related to failure to complete immunization of children aged 10-23 months in Ndoulo (Senegal. Sante. 2009;19:9-13. 19801345 10.1684/san.2009.0139

[19] Mutua MK Kimani-Murage E Ettarh RR Childhood vaccination in informal urban settlements in Nairobi, Kenya: who gets vaccinated? BMC Public Health. 2011;11:6. 10.1186/1471-2458-11-6 21205306 PMC3024932

[20] Tadesse H Deribew A Woldie M Predictors of defaulting from completion of child immunization in south Ethiopia, May 2008: a case control study. BMC Public Health. 2009;9:150. 10.1186/1471-2458-9-150 19463164 PMC2694177

[21] Russo G Miglietta A Pezzotti P Biguioh RM Bouting Mayaka G Sobze MS Vaccine coverage and determinants of incomplete vaccination in children aged 12-23 months in Dschang, West Region, Cameroon: a cross-sectional survey during a polio outbreak. BMC Public Health. 2015;15:630. 10.1186/s12889-015-2000-2 26156158 PMC4496879

[22] Sadoh AE Eregie CO Timeliness and completion rate of immunization among Nigerian children attending a clinic-based immunization service. J Health Popul Nutr. 2009;27:391-5. 10.3329/jhpn.v27i3.3381 19507754 PMC2761795

[23] World Health Organization. Immunization in practice: a practical guide for health staff - 2015 update. Geneva, Switzerland: WHO; 2015.

[24] Wallace AS Willis F Nwaze E Dieng B Sipilanyambe N Daniels D Vaccine wastage in Nigeria: An assessment of wastage rates and related vaccinator knowledge, attitudes and practices. Vaccine. 2017;35 48 Pt B:6751-8. 10.1016/j.vaccine.2017.09.082 29066189 PMC5771486

[25] Wallace AS Krey K Hustedt J Burnett E Choun N Daniels D Assessment of vaccine wastage rates, missed opportunities, and related knowledge, attitudes and practices during introduction of a second dose of measles-containing vaccine into Cambodia’s national immunization program. Vaccine. 2018;36:4517-24. 10.1016/j.vaccine.2018.06.009 29907485 PMC6032508

[26] Oku A Oyo-Ita A Glenton C Fretheim A Ames H Muloliwa A Perceptions and experiences of childhood vaccination communication strategies among caregivers and health workers in Nigeria: A qualitative study. PLoS One. 2017;12:e0186733. 10.1371/journal.pone.0186733 29117207 PMC5678719

[27] Ames HMR Glenton C Lewin S Parents’ and informal caregivers’ views and experiences of communication about routine childhood vaccination: a synthesis of qualitative evidence. Cochrane Database Syst Rev. 2017;2:CD011787. 10.1002/14651858.CD011787.pub2 28169420 PMC5461870

