# Supplementary material for: Access, demand, and utilization of childhood immunization services: A cross-sectional household survey in Western Area Urban district, Sierra Leone, 2019
Source: J Glob Health. 2020 May 15;10(1):010420. doi: 10.7189/jogh.10.010420 (PMC7243070; doi:10.7189/jogh.10.010420)
Supplement: Online Supplementary Document [file jogh-10-010420-s001.pdf]

Supplementary Materials

Appendix S1. Household enumeration and survey sampling methodology:

The household line listing was created during October-December 2018 through enumeration of all structures in each selected EA with information documented on the number of structures, presence and the number of households within each structure, the number of household members, age and sex of each member. Three attempts were made to enumerate each household. Listing completion in each EA was determined using a threshold of 80%.

A sample size of 400 (200 households in slums and 200 households in non-slums) was determined to be sufficient to capture an expected prevalence of 10% of enrolled children who are not up-to-date on the recommended childhood vaccines in each setting, with a precision of 7% when sampling 29 enumeration areas. A non-response rate of 15% and a design effect of 1.6 was assumed.

Table S1. Household level demographic and socioeconomic characteristics (unweighted), household survey, WAU district, Sierra Leone, 2019

Household level characteristics	Slum (n=236)		Non-slum (n=207)	
	n or (median)	[Range] or %	n or (median)	[Range] or %
**Main source of drinking water**				
Piped water	178	75	107	52
Dug well	17	7	46	22
Water from spring	1	<1	6	3
Surface water (river/dam)	0	0	5	2
Water tank	3	1	1	<1
Purchase filtered water	38	16	42	20
**Location of water source**				
In home	11	6	14	8
In own yard/plot	44	22	29	18
Elsewhere	143	72	122	74
**Median time to reach water source (minutes)**	(15)	[1-180]	30	[1-360]
**Type of toilet**				
Flush or pour flush toilet	74	31	74	36
Pit latrine	106	45	123	59
Bucket/ hanging toilet/ plastic bags	56	24	10	5
**HHs possessing all of the following: electricity, radio, television, mobile phone, and refrigerator**	69	29	88	43
**HHs possessing all of the following: rudimentary or finished walls, floors, and roof**	213	89	207	94

Table S2. Caregiver factors and attitudes associated with utilization of routine immunization comparing slum to non-slum areas (weighted), household survey, Western Area Urban district, Sierra Leone, 2019

	Slum (n=232*)		Non-slum (n=210)	
	%	95% CI	%	95% CI
**Transportation time from home to vaccination site**				
<30 minutes	53	45-62	58	45-69
30 minutes – 1 hour	38	30-46	32	24-42
>1 hour	8	4-14	9	5-15
**Perception of time to reach usual vaccination site**				
About right	25	19-31	24	17-32
A short time	41	33-40	36	26-47
Too much time	34	24-45	40	32-50
**Preferred location of additional vaccination site**				
Close to home	88	82-92	90	83-94
Close to work	2	1-5	1	0-2
Close to market	7	4-12	5	3-9
No preference	3	1-9	5	2-9
**Wait time at vaccination site**				
<30 minutes	21	17-25	20	14-29
30 minutes – 1 hour	39	32-47	35	26-44
>1 hour	40	32-47	44	37-52
**Perception of wait time at vaccination site**				
About right	49	39-58	43	35-51
A short time	37	26-40	35	27-45
Too much time	14	10-19	22	16-29
**Payment to healthcare worker**				
Nothing	47	36-58	50	41-58
1,000-5,000 Leones	41	32-52	38	28-49
6,000-10,000 Leones	8	5-13	9	6-14
>10,000 Leones	4	1-8	3	1-7
**Knowledge of child in family or community who experienced vaccine side effects within last 12 months**	7	4-13	7	4-13
**Self-reported refusal of recommended vaccination**	6	3-12	2	1-5
**Self-reported delays in receiving recommended vaccination**	36	26-46	37	28-48
**Would vaccinate future child with all recommended vaccines**	99	97-100	99	95-100
**Attended vaccination site but child did not receive vaccination**	13	8-18	10	6-15
**Reason for attending but not receiving**	**N=28**		**N=23**	
Not enough children to open a vaccination vial	26	14-43	48	25-72
Vaccines not available	25	12-44	6	1-22
Not a scheduled vaccination day	34	15-59	29	10-61
Other	15	6-33	17	5-45
Wait time was too long, chose not to go through with vaccination or asked to pay or tip for service	0	-	0	-

*Missing data from two caregivers

Note: No significant differences at $p<0.05$ level.

Table S2. Caregiver reason for refusal or delay of recommended vaccination (unweighted), household survey, WAU district, Sierra Leone, 2019

**Reason for self-reported refusal***	**Slum (n=13) n (%)**	**Non slum (n=4) n (%)**
Fear of vaccination side effects	9 (69)	1 (25)
Lack of trust in vaccinator	2 (15)	1 (25)
No particular reason	1 (8)	1 (25)
Lack of trust in vaccine	1 (8)	0 (0)
Fertility concerns	0 (0)	1 (25)
Child or caregiver was ill	1 (8)	0 (0)
Other (e.g., fear of hurting child during vaccination)	1 (8)	0 (0)
Lack of trust in health system, too many vaccinations in one visit, lack of benefit to vaccine, concerns about cost or religion	0 (0)	0 (0)
**Reason for self-reported delay***	**Slum (n=83)**	**Non slum (n=80)**
Lack of time to take child	35 (42)	40 (50)
Child or caregiver was sick	16 (19)	12 (15)
Fear of vaccination side effects	11 (13)	5 (6)
Other (e.g., inclement weather or holiday)	6 (8)	9 (11)
Forgot about vaccination	8 (10)	5 (6)
No particular reason	5 (6)	4 (5)
Long distance to get to vaccination site	2 (2)	4 (5)
Lack of funds to pay the tip/token of appreciation	3 (4)	1 (1)
Long waiting time at vaccination site	2 (2)	1 (1)
Negative experiences with healthcare providers	2 (2)	1 (1)
Lack of trust in vaccinator	2 (2)	1 (1)
Administering too many vaccines during the same visit	0 (0)	2 (3)
Lack of funds to pay for transportation	2 (2)	0 (0)
Lack of trust in vaccine	1 (1)	0 (0)
Misplaced vaccination card	1 (1)	4 (5)
Lack of benefit to vaccinate, lack of trust in health system, religious or fertility concerns	0 (0)	0 (0)

*Categories are not mutually exclusive

Table S4. Caregiver perceptions related to childhood vaccinations comparing slum to non-slum areas and receiving three doses of pentavalent vaccine among children who had a vaccination card at the time of interview (weighted), household survey, Western Area Urban district, Sierra Leone, 2019

**Perceptions of vaccination (% very much)***	**Slum N=233****	**Non-slum N=210**	**P-value**	**Received three pentavalent vaccine doses N=309**	**Did not received three pentavalent vaccine doses N=41**	**P-value**
Vaccines are good for your child	98 (95-99)	100 (98-100)	**0.04**	99 (98-100)	100	0.69
Vaccines are safe for your child	91 (86-94)	90 (83-94)	0.86	90 (85-94)	64 (21-92)	0.08
Vaccines protect your child against diseases	95 (81-97)	99 (96-100)	**0.04**	98 (95-99)	100 (96-100)	0.14
Confident in your ability to take your child for vaccination visit	88 (82-93)	92 (84-96)	0.38	94 (89-97)	64 (21-93)	**0.02**
Encourage others to get their children vaccinated	67 (61-73)	73 (64-81)	0.25	71 (62-79)	44 (16-77)	0.09
People in your community value childhood vaccination services	74 (66-81)	82 (73-88)	0.15	78 (69-86)	47 (17-79)	**0.03**
Your spouse, partner or other family member approves of vaccinations	88 (83-92)	94 (88-97)	0.07	95 (91-98)	53 (19-84)	**<0.001**
Other parents in your community approve of childhood vaccinations	69 (61-76)	77 (68-84)	0.17	76 (67-83)	53 (31-74)	**0.01**
Trusted leaders in your community approve of childhood vaccinations	75 (68-81)	74 (65-82)	0.84	75 (67-82)	68 (49-82)	0.31
Measles is a health threat for children who are unvaccinated	92 (88-95)	94 (88-97)	0.50	94 (86-98)	94 (80-98)	0.85
Illnesses prevented by vaccinations are severe	90 (86-94)	95 (90-97)	0.06	94 (90-97)	95 (85-98)	0.88

*Comparison is between the "very much" response option and "somewhat", "very little", and "not at all" combined.
**Missing data from one caregiver

Table S5 . Vaccination coverage of children ages 12-23 months and characteristics (weighted), household survey, Western Area Urban district, Sierra Leone, 2019

**Vaccination coverage**	**12-23 months old (N=209)**	
	% or (mean)	95% CI
**Mean age of most recent vaccination in months**	11	11-12
**Owns vaccination card**		
Yes, available	82	75-87
Yes, not seen	11	7-18
No	7	3-15
**Vaccination coverage (by card or caregiver recall)**		
BCG vaccination	100	100
Penta1	100	100
Received two doses of penta	100	100
Received all three penta doses	100	100
MCV1	82	73-89
MCV2[¥]	30	21-39
Received BCG, all penta doses and 2 measles doses[¥]	23	15-33
Received BCG, all penta doses and 2 measles doses by 24 months[¥]	19	14-27
No vaccinations	0	0

Abbreviations: BCG = Bacillus Calmette-Guérin; Penta = pentavalent vaccine (diphtheria-tetanus-pertussis-hepatitis B-*Haemophilus influenzae* type b); MCV1 = measles-containing vaccine, 1st dose; MCV2 = measles-containing vaccine, 2nd dose

[¥]Only children ≥15 months were included in the analysis, n=168

Appendix S2. Household Survey Questionnaire – Urban Needs Assessment

Date	|___||___| / |___||___| / |___||___|*(dd/mm/yy)*
Team ID	
Enumerator ID	
Enumeration area / Cluster	
Household ID	
Infant ID	
Ward	1. West I<br>2. West II<br>3. West III<br>4. Central I<br>5. East I<br>6. East II<br>7. East III
Number of visits made	1. One Date of visit: _________________<br>2. Two Date of visit: _________________
Can data be collected for the household?	☐1. Yes<br>☐2. No

1. Outcome of visit	Visit 1	Visit 2
Completed	1	1
No HH member at home/no competent respondent	2	2
Entire HH absent for extended period	3	3
Postponed	4	4
Refused to be interviewed	5	5
Dwelling vacant/address not a dwelling	6	6

Dwelling destroyed	7	7
Dwelling not found	8	8
Deaf/Did not speak a survey language	9	9
No eligible respondents in household	10	10
Interview partially completed	11	11
Entire HH moved out of EA	12	12
Eligible caregiver moved out of EA	13	13
Eligible infant moved out of EA	14	14
Other (specify)__________________________	15	15

START_TIME (for tablet)

100	Eligibility and Consent		
ID	QUESTION	RESPONSE	NOTES / SKIP PATTERN
101.	How many children 12-36 months of age live in this household **for which you are the primary caregiver**?<br><br>*ONLY enter number of children for which the respondent is the primary caregiver.*<br><br>***(armos pikin dem wae dae between 12 to 36 month dae na dis pot wae na u dae take care of dem?)***	|____| |____| <br>Enter 99 if don't know	This question is just to confirm that there are indeed eligible children in the household based on the listing<br><br>If 0 then tablet will show a message to stop and talk to supervisor<br><br>Limit total to 5 eligible children
102.	Write the name of each child age 12-36 months of age in the box to the right.	1. Name:<br>2. Name:<br>3. Name:<br>4. Name:	

Informed Consent Statement<br><br>Thank you for your time. We are here today representing Sierra Leone Ministry of Health and its partners. We would like to ask you about your experience with immunizations that your child aged 12-36 months has received. The information provided will help the Ministry in improving the country's immunization programme. The information we collect will be anonymous, which means that you and your child/children will not be personally identified with the information. The interview should take 30-45 minutes to conduct. Participation in the survey is voluntary, but I hope you will agree to answer the questions since your views are important.			
103.	Do you consent to participate in the interview?<br>***(u gree for leh we tok?)***	☐1. Yes<br>☐2. No	If NO, thank the person for his/her time and continue to the next household.
Complete these questions for ALL children between 12 and 36 months old for which this respondent is the primary caregiver			
104.	Is the respondent 15 years of age or older?<br>***(u don 15 year or u don pass?)***	☐1. Yes<br>☐2. No	If NO, thank the respondent for their time and schedule a revisit when an adult will be available and continue to the next household.
105.	Are you the primary caregiver of this child?<br>***(Na u na d main porsin wae dae take care of dis pikin?)***	☐1. Yes<br>☐2. No ----------------------------------→	If NO, thank the respondent for their time and schedule a revisit when primary caregiver will be available and continue to the next household.

106.	Date of birth of (NAME)<br>***(wus tem (NAME) bon?)***	Day (DD) |____| |____| <br>Month (MM) |____| |____| <br>*99 = don't know*<br><br>Year<br><br>☐ 2016<br>☐ 2017<br>☐ 2018<br>☐ Don't know	If there are no other eligible children in household, thank the respondent and continue to the next household.

	**Family and Child Demographic Characteristics**<br><br>I would now like to ask you a few questions about your home.<br><br>NB: Ask child's name and substitute "this child" with the name of the child in this section.		
**ID**	**QUESTION**	**RESPONSE**	**GO TO**
201.	How many people live in this household as primary residents?<br><br>***(armos people dem gbain dae live na dis hos wae na wuna all dae eat na d same pot?)***<br><br>*(defined as those who regularly sleep in the household and live there the majority of the past year)*	|____| |____|	

202.	What is your relationship to (NAME)?<br><br>*(U Na watin to (NAME)?)*	☐1. Mother<br><br>☐2. Father<br><br>☐3. Grandmother<br><br>☐4. Grandfather<br><br>☐5. Other guardian / caregiver<br><br>Specify________________________	1→ 204
203.	Is the mother of (NAME) still living?<br><br>*((NAME) ein Mama dae alive?)*	☐1. Yes<br>☐2. No	
204.	How many children did (NAME)'s mother give birth to altogether, including those who died (if any) after birth and those who are no longer living with you?<br><br>***(Armos pikin dem (NAME) ein mama don born, weda e don die or e nor dae witam na ya?)***	|____| |____| children<br><br>*If don't know, enter 99*	
205.	How many living children does (NAME)'s mother have in total?<br><br>*(armos pa den wanya dae alive?)*	|____| |____| children<br><br>*If don't know, enter 99*	
206.	Starting from the oldest child, what birth order is (NAME), including those who have died or are no longer living with you?<br>***(starting from d fors pikin(NAME) na de number armos pikin wae e bon?)***	|____| |____| rank	
207.	What is (NAME)'s sex?<br><br>*((NAME) Na man or woman?)*	☐1. Female<br><br>☐2. Male	

208.	Where was (NAME) born?<br><br>***(Wusai den bon (NAME)?)***	☐1. Home<br><br>☐2. Health facility (any type)<br><br>☐3. Traditional birth attendant (TBA) site<br><br>☐4. Other, specify<br><br>☐99. Don't know	
209.	How long have you lived in this neighborhood?<br><br>***(aw long u don tap na dis area?)***<br><br>*If less than 1 year, please include number of months. If more than 1 year, round to the nearest year and put "0" for months.*	|____| |____| years<br><br>or if less than 1 year:<br><br>|____| |____| months<br><br>*99 if don't know*	IF Q203 = NO, SKIP TO 215
210.	What is (NAME)'s mother's marital status?<br><br>***((NAME) e mama marade?)***	☐1. Married<br><br>☐2. Living together<br><br>☐3. Divorced or separated<br><br>☐3. Widowed<br><br>☐4. Never married and never lived together	
211.	What is (NAME)'s mother's age?<br><br>***(Armos year (NAME) e mama old?)***	|____| |____| Years<br><br>*99 if don't know*	

212.	What is (NAME)'s mother's highest level of school completed?<br><br>***(wusai (NAME) e Mama tap pa book learning?)***	☐1. Never attended school/no formal education<br><br>☐2. Attended some primary school<br><br>☐3. Primary<br><br>☐4. Junior secondary<br><br>☐5. Senior secondary<br><br>☐6. Vocational / tertiary<br><br>☐7. Higher<br><br>☐88. Don't Know	
213.	What is (NAME)'s mother's religion?<br><br>***(Watin na (NAME) e mama religion?)***	☐1. Christian<br><br>☐2. Muslim<br><br>☐3. Traditionalist<br><br>☐4. None<br><br>☐5. Other (specify)<br><br>☐88. Don't Know	
214.	What does (NAME)'s mother do as her primary way to earn money?<br><br>***(Watin na de main tin (NAME) e mama dae do for get moni?)***	☐1. Petty trader<br>☐2. Laborer: Plumber/Carpenter/Electrician/Mechanic/Contractor/Fisher man<br>☐3. Private business (**except** petty traders)<br>☐4. Farmer<br>☐5. Teacher/lecturer/Instructor<br>☐6. Public transportation driver (taxi, buses, podapoda)<br>☐7. Okada/ Keke driver<br>☐ 8. Medical or heath professional<br>☐ 9. Soldier<br>☐ 10. Other government employee (**except** soldier)<br>☐ 11. NGO<br>☐ 12. Student<br>☐ 13. Unemployed<br>☐ 14. Retired<br>☐ 15. Other (specify)<br>☐88. Don't Know	
215.	Is the father of (NAME) still living?<br><br>***((NAME) e papa dae alive?)***	☐1. Yes<br>☐2. No---→	Q219

216.	What is (NAME)'s father's religion?<br><br>***(Watin na (NAME) e papa religion?)***	☐1. Christian<br><br>☐2. Muslim<br><br>☐3. Traditionalist<br><br>☐4. None<br><br>☐5. Other (specify)<br><br>☐88. Don't Know	
217.	What is (NAME)'s father's highest level of school completed?<br><br>***(wusai (NAME) e Papa tap pa book learning?)***	☐1. Never attended school/no formal education<br><br>☐2. Attended some primary school<br><br>☐3. Primary<br><br>☐4. Junior secondary<br><br>☐5. Senior secondary<br><br>☐6. Vocational / tertiary<br><br>☐7. Higher<br><br>☐88. Don't know	
218.	What does (NAME)'s father do as his primary way to earn money?<br><br>***(Watin na de main tin (NAME) e papa, dae do for get moni?)***	☐1. Petty trader<br>☐2. Laborer: Plumber/Carpenter/Electrician/Mechanic/Contractor/Fisher man<br>☐3. Private business (**except** petty traders)<br>☐4. Farmer<br>☐5. Teacher/lecturer/Instructor<br>☐6. Public transportation driver (taxi, buses, podapoda)<br>☐7. Okada/ Keke driver<br>☐ 8. Medical or heath professional<br>☐ 9. Soldier<br>☐ 10. Other government employee (**except** soldier)<br>☐ 11. NGO<br>☐ 12. Student<br>☐ 13. Unemployed<br>☐ 14. Retired<br>☐ 15. Other (specify)<br>☐88. Don't Know	

219.	Who holds primary responsibility for household chores such as cooking, cleaning, taking care of family members?<br><br>***(Wudat na d main porsin wae dae take care of d hos woke dem lek for cook,clean en take care of de fambul dem?)***	☐1. Mother of child<br>☐2. Father of child<br>☐3. Child's grandparent<br>☐4. Both mother and father of child<br>☐5. Mother and another family member<br>☐6. Other relatives<br>☐7. Other ________________	
220.	Does someone besides you (neighbor, relative, nanny, etc.) look after/take care of (NAME) for several hours at a time?<br><br>***(Any bodi apart from u lek u neaba or u orda fambul dem or u nanny kin takecare of (NAME) for boku hours any tem e hol lam?)***	☐1. Yes<br>☐2. No	1→221<br><br>2→301
221.	How often does someone besides you look after (NAME) for several hours at a time?<br><br>***(Armos tem dis porsin apart from u kin take care of (NAME)for boku hours any tem e hol lam?)***	☐1. Daily<br>☐2. Weekly<br>☐3. Monthly<br>☐4. Less than once a month	

300	Child's Vaccination History		
ID	QUESTION	RESPONSE	GO TO
301	How old was {NAME} in months at their most recent vaccination visit?<br><br>*(armos month(NAME) bin ol wae e take d last marklate?)*<br><br>*Provide the age in months based on caregiver recall. Do not abstract from the immunization card.*	☐☐ months<br><br>☐ Don't know<br><br>☐ Never vaccinated --→	Q303

302	As far as you know, has your child received all of the recommended immunizations up to the current age of the child?<br><br>*(as far as u no for dis pikin e age, e don take all d marklate wae e suppose for don take ?)*<br><br>*Provide the answer based on caregiver recall. Do not abstract from the immunization card.*	☐1. Yes<br>☐2. No<br><br>☐88. Don't know	
303	Do you have an immunization card [under 5 card] or a card where (NAME)'s vaccinations are written down?<br><br>*(u get marklate card (da yellow wan) or sie wusai den mak all de marklate(NAME) don take?)*<br><br>If YES, May I see it please? *Have the respondent get the immunization card, if available.*<br><br>*(If Yes, ar kin see am)*	☐1. Yes, seen<br><br>☐2. Yes, not seen<br><br>☐3. No card	1→306,<br>3→304

304	Why do you not have an immunization card [yellow card] or other documentation of immunization history for (NAME) today?<br><br>*(watin mek tiday u nor get marklate card, da yellow wan or orda sie wusai den write all d pikin e marklate dem wae (NAME) don take?)*<br><br><u>Do not read responses</u><br><br>*Ask for the main reason and mark ONLY ONE.*	☐1. Child never received an immunization card<br>☐2. Health facility keeps the immunization card<br>☐3. Immunization card is lost or destroyed<br>☐4. Immunization card is with child/kept at a different location<br>☐5. Other (Specify:________________________)<br><br>☐88. Don't know	If no card available, go to Q400. Otherwise continue to Q306.

Review the health card / immunization card of the infant provided by the mother<br>For each vaccine, check « Yes » if there is a checkmark or a date for the vaccine and « no » if there is nothing written. Copy the dates. Check tick box if there is a poorly written or missing day/month/year.						
306	(NAME)'s date of birth (DOB) recorded on card ☐☐ / ☐☐ / ☐☐☐☐<br>a. DD b. MM c. YYYY					
307	Type of card(s)<br><br>*Mark all available.*		☐1. Child Health Record Book------→<br>☐2. Under 5 card<br>☐3. Piece of paper/other documentation---------------------------→			Q309<br><br>Q309
308	Does the yellow card have designated space to document IPTi doses (new yellow card)?		☐1. Yes<br>☐2. No			
	**Vaccine**	**a. Vaccine received**	**b. Day**	**c. Month**	**d. Year**	**e. Date poorly written or missing day/month/year**
309	BCG	☐1. Yes<br>☐2. No	☐☐	☐☐	☐☐☐☐	☐

310	Pentavalent (1) DTP/Hib/HepB	☐1. Yes<br>☐2. No	☐☐	☐☐	☐☐☐☐	☐
311	Pentavalent (2) DTP/Hib/HepB	☐1. Yes<br>☐2. No	☐☐	☐☐	☐☐☐☐	☐
312	IPTi 1 (at 10 weeks)	☐1. Yes<br>☐2. No<br>☐3. Not recorded/unknown	☐☐	☐☐	☐☐☐☐	☐
313	Pentavalent (3) DTP/Hib/HepB	☐1. Yes<br>☐2. No	☐☐	☐☐	☐☐☐☐	☐
314	IPTi 2 (at 14 weeks)	☐1. Yes<br>☐2. No<br>☐3. Not recorded/unknown	☐☐	☐☐	☐☐☐☐	☐
315	Measles (1)	☐1. Yes<br>☐2. No	☐☐	☐☐	☐☐☐☐	☐
316	IPTi 3 (at 9 months)	☐1. Yes<br>☐2. No<br>☐3. Not recorded/unknown	☐☐	☐☐	☐☐☐☐	☐

317	Measles (2)	☐1. Yes<br>☐2. No	☐☐	☐☐	☐☐☐☐	☐
318	Insecticide-treated bednet received	☐1. Yes<br>☐2. Info not recorded	☐☐	☐☐	☐☐☐☐	☐
319	Photo of immunization dates from immunization card<br><br>**Take photo of the vaccination dates on the immunization card. DO NOT capture *Child Health Card Personal Information* in your photo.**					

**400**	**Recall of Child's Vaccination History**<br><br>*Only when vaccination card not available*<br><br>*Otherwise if card available and vaccination dates extracted, then go to →Q407*		
**ID**	**QUESTION**	**RESPONSE**	**GO TO**
401	Has the child ever received an injection in the right upper arm or shoulder that usually causes a scar? – that is, BCG vaccination (against tuberculosis). **This is usually given at birth or very shortly after.**<br>***(Den don ever chuke u pikin up e right hand or shoulder wae e kin lef mark dae? ,"that na BCG marklate against dri kof. d fos marklate wae den dae gee pikin as den bon am)***	☐1. Yes ---→<br>☐2. No --→<br>☐3. Don't know--→	Q402<br>Q403<br>Q403
402	If the child is present, check for evidence of a scar and record	☐1: Scar Present<br>☐2: No Scar Present<br>☐3: Child not available to check	
403	Has the child ever received an injection on the left thigh? – that is a five-in-one vaccination (pentavalent) to prevent him/her from getting tetanus, whooping cough, diphtheria, influenza & hepatitis. **It is usually given to the child at 6 weeks, 10 weeks, and 14 weeks of age.**<br>***(den don ever chuke u pikin up e left leg ?) (dat na da five in wan marklate wae dae hep pikin for leh e nor get tetanus,kof en orda sik dem)***	☐1. Yes ---→<br>☐2. No --→<br>☐3. Don't know ---→	Q404<br>Q405<br>Q405

404	How many times did the child receive pentavalent vaccine on the left thigh?<br>*(Armos tem dis pikin don take da five in wan marklate na e left leg?)*	Number of times: __________<br>*Enter 99 if don't remember / unsure*	
405	Has the child ever received an injection on the **upper arm**? that is measles injection at the **age of 9 months or older** - to prevent him/her from getting measles<br>***(den don ever chuke u pikin up e hand da measle injection wae den dae gee am wae e 9 month for mek e nor go get measles ?)***	☐1. Yes --→<br>☐2. No --→<br>☐3. Don't know --→	Q406<br>Q407<br>Q407
406	How many times was measles vaccine given at a routine immunization session?<br>***(armos tem den gee am mealse marklate d tem dem wae u bi dea kerr am go for marklate?)***	Number of times: __________	
407	Has (NAME) ever taken the new medication that helps prevent him/her from getting malaria?<br>***((NAME) be don ever take da new meresin wae go hep am for mek e nor get malaria?)***	☐1. Yes<br>☐2. No<br>☐3. Don't Know	If *2,3*→ *500*
408	How many times was (NAME) given the new medication to prevent him/her from getting malaria?<br>***Armos tem (NAME) don take da new meresin wae go hep am for mek e nor get malaria?***	☐1. One<br>☐2. Two<br>☐3. Three<br>☐4. More than Three<br>☐5. Don't Know	

409	Approximately how long did you have to wait at the health facility to receive the medication that prevents malaria in infants?<br>***(Lek how long u get for wait na d hospitul or clinic for get d meresin wae dae make pikin dem nor get malaria?)***	☐1. Less than 30 minutes<br>☐2. Between 30 minutes and 1 hour<br>☐3. Over 1 hour<br>☐77. Unsure of time	
410	Did (NAME) have any reactions after taking the new medication helping to prevent him/her from getting malaria?<br>***(any tin be happen to (NAME) after wae e take da meresin wae dae hep am for mek e nor get malaria?)***	☐1. Yes<br>☐2. No<br>☐3. Don't Know	If *2,3, 99→ 501*
411	What kind of reaction(s) did {NAME} have?<br>***(Wus kin tin dem happen to ram?)***<br>*Mark all that apply*	☐1. Skin rash<br>☐2. Vomiting<br>☐3. Diarrhea<br>☐4. Fever<br>☐5. Other__________________________<br>(SPECIFY)	
412	How long did this reaction last after (NAME) took the medication?<br>***(aw long dis tin ya bin last for after (NAME) don take dis new malaria Meresin?)***<br>*If more than one reaction, enter the number of days for the reaction that lasted the longest period of time*	NUMBER OF DAYS<br>☐☐<br>*Enter 88 if don't know*<br>*Enter 99 if declined to answer*	
413	Did you see a health care provider about the reactions (NAME) had after taking the new malaria prevention medication?<br>***(u bi go meet welbodi woke man bot d tin dem way happen to (NAME) after wae e take da new meresin wae dae mek porsin nor get Malaria?)***	☐1. Yes<br>☐2. No<br>☐3. Don't Know	

500	Availability and Accessibility of Childhood Vaccination Services		
ID	QUESTION	RESPONSE	GO TO
501	Where did (NAME) receive their most recent immunization?<br>***(wusai (NAME) get e last Marklate?)***	☐1. Public / Government<br>☐2. Private (non-NGO)<br>☐3. NGO<br>☐4. Mission<br>☐5. At home during outreach------------------------------ ><br>☐6. School-- ><br>☐ 7. N/A: Child never vaccinated-------------------------------→<br>☐88. Don't know / don't remember---------------------- >	 <br><br><br>Q503<br>Q503<br>Q510<br>Interviewer should show facility list to help elicit response
502	What was the name of the health facility where (NAME) receive their most recent immunization?<br>***(watin na d name of d hospital or clinic wae (NAME) take e last marklate?)***	Dropdown list of ALL health facilities, organized by type (categories above). Include option for "other, specified", "Don't know" and "outside of Western Area Urban"	
503	Has (NAME) always gone for routine immunization at the same site?<br>***((NAME) kin always go for ein marklate dem na de same sie?)***	☐1. Yes --- ><br>☐2. No<br>☐99. Don't know -- >	Q505<br><br>Q505

504	What is the main reason why you did not go to the same site?<br><br>*(watin na d main tin wae make u nor go na d same sie?)*<br><br><u>Do not read responses</u><br><br>*Ask for the main reason and mark ONLY ONE*	☐1. Family moved<br>☐2. Site (health facility or outreach) closed or moved<br>☐3. Availability of vaccine<br>☐4. Instructed by health care worker<br>☐5. Convenience of location<br>☐6. Other personal preference<br>☐7. Too expensive (facility charges too much)<br>☐8. Lack of funds for transport<br><br>☐9. Other __________________	
505	Approximately, how long from home does it take to get to your usual vaccination site using your usual type of transportation?<br>*(lek how long e go take u for komot na hos en go na d sie wae den dae gee marklate if u use d way u kin go dae?)*	☐1. Less than 30 minutes<br>☐2. Between 30 minutes and 1 hour<br>☐3. Over 1 hour<br><br>☐77. Unsure of time---→<br>☐88. Unsure of location of vaccination site------------→	 <br><br><br>Q507<br>Q507
506	How do you view the time it usually takes to reach your usual vaccination site?<br>*(how u see da tem wae u kin take for go na d sie way u kin go for marklate?)*	☐1. Too much time<br>☐2. About right<br>☐3. A short time	
507	Once you are at the vaccination site, approximately how long does it usually take to get (NAME) vaccinated at the site?<br>*(wae u don reach na d sie wae den dae gee marklate,how long e kin take for mek den gee (NAME) Marklate?)*	☐1. Less than 30 minutes<br>☐2. Between 30 minutes and 1 hour<br>☐3. Over 1 hour<br><br>☐77. Unsure of time ---------------------------------------→	 <br><br><br>Q509

508	How do you view the time it usually takes to get your child vaccinated once you arrive at the vaccination site?<br>***(wae u don reach na d sie wae den dae gee marklate,how u see da tem wae e kin take for mek den gee u pikin Marklate?)***	☐1. Too much time<br>☐2. About right<br>☐3. A short time	
509	How much are you usually expected to give to healthcare workers during a vaccination visit?<br>***(armos u feel say u for gee welbodi woke man dem, d tem wae u kerr u pikin for marklate?)***<br><br>*Probe to understand payments or gifts that are not required but expected.*	☐1. Nothing<br>☐2. 1,000-5,000 Leones<br>☐3. 6,000-10,000 Leones<br>☐4. >10,000 Leones<br>☐5. Non-monetary gift<br><br>☐88. Don't remember / unsure	
510	Have you ever taken any of your children for vaccination and come home without receiving the vaccination?<br>***(u don ever kerr any of u pikin dem for marklate en u return back without den nor gee am Marklate?)***	☐1. Yes--→<br>☐2. No--→<br>☐88. Don't remember / unsure--------------------------→	Q511<br>Q601<br>Q601
511	What was the main reason any of your children did not receive vaccination the last time that happened (*referring to Q510*)?<br>***(watin na d main reason wae den nor gee any wan pa u pikin dem marklate de last tem way u kerram go?)***	☐1. Vaccines were not available<br>☐2. Was not a scheduled vaccination day<br>☐3. The wait was too long<br>☐4. I was asked to pay for service or related materials<br>☐5. I was asked to provide a tip/token of appreciation<br>☐6. I chose not to go through with vaccination<br>☐7. Not enough children to open a vaccination vial at that time<br>☐8. Other (specify:_________________________)<br><br>☐88. Don't remember / unsure	

The next several questions are about your opinion on vaccination. For every question that I ask, please look at this figure. The biggest circle means "very much", the second circle means "somewhat", the small circle means "very little" and no circle means "not at all". Please point to the circle that relates to how much you feel about every question.

(D Next question dem wae ar dae kam ask,na watin u think bot vaccination.any question wae ar ask du ya ,luk pa dis picture. De big circle mean Boku Boku wan ,de second circle mean,haf en haf,de small circle mean small ,en d sie wae u nor dae see circle mean,Natin . Duya point pa d circle u tink sae feba u answer)

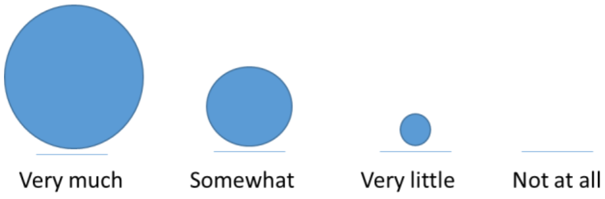

600	Vaccination Acceptance and Demand		
ID	QUESTION	RESPONSE	GO TO
601	How much do you think that vaccines are good for your child?<br><br>***(armos u tink say marklate good for u pikin?)***<br><br>*Note: Point to the visual scale, and ask which one of the circles represent how much he/she thinks vaccines are good*<br><br>***(Lek dis much, or dis much, or dat much? Ose wan wae dae show armos u think say marklet good…)***	☐ 1. Very much<br>☐ 2. Somewhat<br>☐ 3. Very little<br>☐ 4. Not at all<br><br>☐ 99. Declined to answer	
602	How much do you think that vaccines are safe for your child?<br><br>**(Armos u tink say marklet na tin wae safe for u pikin?**	☐ 1. Very much<br>☐ 2. Somewhat<br>☐ 3. Very little<br>☐ 4. Not at all<br><br>☐ 99. Declined to answer	
603	How much do you think that vaccines protect your child against diseases?<br><br>***(armos u think say marklate dae hep u pikin for mek e nor get sik dem?)***	☐ 1. Very much<br>☐ 2. Somewhat<br>☐ 3. Very little<br>☐ 4. Not at all<br><br>☐ 99. Declined to answer	
604	How much do you feel confident in your ability to take your child for scheduled vaccination visits?<br><br>***(Armos u feel say u go always able for take you pikin for all e maklate dem wae di tem reach?)***	☐ 1. Very much<br>☐ 2. Somewhat<br>☐ 3. Very little<br>☐ 4. Not at all<br><br>☐ 99. Declined to answer	
605	How much would you encourage others to get their children vaccinated?<br><br>***(armos u able encourage orda combra dem for take dem pikin go for marklate?)***	☐ 1. Very much<br>☐ 2. Somewhat<br>☐ 3. Very little<br>☐ 4. Not at all<br><br>☐ 99. Declined to answer	

606	How much do people in your community value childhood vaccination services?<br><br>***(Armos u tink say pipul dem na u area really see say e impotant for marklate pikin dem?)***	☐ 1. Very much<br>☐ 2. Somewhat<br>☐ 3. Very little<br>☐ 4. Not at all<br><br>☐ 99. Declined to answer	
607	How much does your spouse, partner, or other family members in your household approve of childhood vaccination?<br><br>***(Armos u tink say u man (uman) or other family member dem na di same pot go gree for gee pikin marklate?)***	☐ 1. Very much<br>☐ 2. Somewhat<br>☐ 3. Very little<br>☐ 4. Not at all<br><br>☐ 99. Declined to answer	
608	How much do other parents in your community approve of childhood vaccination?<br><br>***(Armos u tink say orda combra dem na u area go gree for gee pikin marklate?)***	☐ 1. Very much<br>☐ 2. Somewhat<br>☐ 3. Very little<br>☐ 4. Not at all<br><br>☐ 99. Declined to answer	
609	How much do trusted leaders in your community approve of childhood vaccination?<br><br>***(Armos u think say d leaders dem (or big wan dem) wae pipul kin listen to na una area, kin da gree for make pikin dem take marklate?)***	☐ 1. Very much<br>☐ 2. Somewhat<br>☐ 3. Very little<br>☐ 4. Not at all<br><br>☐ 99. Declined to answer	
610	How much does your religion influence vaccination decisions for your child?<br><br>**(Armos u tink say u religion kin dae affect u decision for take the pikin go for marklate?)**	☐ 1. Very much<br>☐ 2. Somewhat<br>☐ 3. Very little<br>☐ 4. Not at all<br><br>☐ 99. Declined to answer	

611	How much of a health threat do you think measles is for children who are unvaccinated?<br><br>***(Armos u tink say measles go hamgborg pikin ein wellbodi wan wae nor take marklate?)***	☐ 1. Very much<br>☐ 2. Somewhat<br>☐ 3. Very little<br>☐ 4. Not at all<br><br>☐ 99. Declined to answer	
612	How much would you say childhood vaccination goes together with your religious beliefs?<br><br>***(armos u tink u religion gree for pikin marklate?)***	☐ 1. Very much<br>☐ 2. Somewhat<br>☐ 3. Very little<br>☐ 4. Not at all<br><br>☐ 99. Declined to answer	
613	How much do you think the illnesses which vaccination prevent are severe?<br><br>***(Armos u tink say small-pikin sickness dem (lek measles and polio) go cause serious problem for pikin dem?)***	☐ 1. Very much<br>☐ 2. Somewhat<br>☐ 3. Very little<br>☐ 4. Not at all<br><br>☐ 99. Declined to answer	

For next set of questions (700-717) you may say "yes" "unsure" or "no" to tell us about your observations and experiences with childhood vaccination.

700	Vaccination Experiences and Behaviors		
ID	QUESTION	RESPONSE	GO TO
701	Do you know of any child in your family or community that has experienced vaccine side effects in the last year?<br><br>***(u sabie any pikin na u family or na u area wae marklate don hamborg insie dis pass one year?)***	☐ 1. Yes---→<br>☐ 2. No--→<br>☐ 3. Unsure ---→<br><br>☐ 99. Declined to answer	Q702<br><br>Q703<br><br>Q703
702	How serious was the vaccine side effect?<br><br>***(how dis tin ya be muna ram?)***	☐ 1. Not at all serious<br>☐ 2. A little serious<br>☐ 3. Somewhat serious<br>☐ 4. Very serious<br><br>☐ 99. Declined to answer	

703	Have you ever refused getting a recommended vaccine for your child? By refused, I mean, have you ever turned down vaccination services or chosen not to have your child vaccinated?<br><br>***(u don ever deny marklate way u pikin for get?)***	☐ 1. Yes--→<br>☐ 2. No--→<br>☐ 3. Unsure---→<br><br>☐ 99. Declined to answer --------------------------------→	Q704<br><br>Q705<br><br>Q705<br><br>Q705
704	What was the reason for refusing the recommended vaccine(s) for your child?<br><br>***(for wus reason wae mek u deny marklate way u pikin for get?)***<br><br>(multiple selections allowed; do not read)<br><br><u>*PROBE: Any other reasons*?</u>	☐ 1. Lack of trust in vaccine<br>☐ 2. Lack of trust in vaccinator<br>☐ 3. Lack of trust in health system<br>☐ 4. Administering too many vaccines in the same visit<br>☐ 5. Fear of vaccination side effects or past experience with vaccination side effects<br>☐ 6. Concerns about cost<br>☐ 7. Religious concerns<br>☐ 8. Fertility concerns<br>☐ 9. Lack of benefit to vaccinate<br>☐ 10. Negative experiences with healthcare providers<br>☐ 11. Other (specify)<br>☐ 12. No particular reasons<br><br>☐ 88. Don't remember why<br>☐ 99. Declined to answer	
705	Have you ever delayed getting a recommended vaccine for your child?<br><br>***(u don ever miss d tem for u pikin for take marklate?)***	☐ 1. Yes--→<br>☐ 2. No--→<br>☐ 3. Unsure ---→<br><br>☐ 99. Declined to answer --------------------------------→	Q706<br><br>Q707<br><br>Q707<br><br>Q707

706	What was the reason or barriers for delaying the recommended vaccine(s) for your child?<br><br>***(watin na d tin dem wae mek ,u miss d tem wae u pikin for take marklate?)***<br><br>(multiple selections allowed; do not read)<br><br><u>*PROBE: Any other reasons*</u>?	☐ 1. Lack of trust in vaccine<br>☐ 2. Lack of trust in vaccinator<br>☐ 3. Lack of trust in health system<br>☐ 4. Administering too many vaccines in the same visit<br>☐ 5. Fear of vaccination side effects or past experience with side effects<br>☐ 6. Long waiting time at vaccination site<br>☐ 7. Long distance to get to vaccination site<br>☐ 8. Lack of time to take the child (working, travel, other priorities)<br>☐ 9. Lack of funds to pay for transportation<br>☐10. Lack of funds to pay the tip/token of appreciation for providers<br>☐ 11. Religious concerns<br>☐ 12. Fertility concerns<br>☐ 13. Lack of benefit to vaccinate<br>☐ 14. Negative experiences with healthcare providers<br>☐ 15. Other (specify)<br>☐ 16. No particular reasons<br><br>☐ 88. Don't remember why<br>☐ 99. Declined to answer	
707	If you have another child in the future, do you plan to accept ALL recommended vaccinations for him/her?<br><br>***(if u get orda pikin tumara,u go plan for leh e get ol d marklate wae e suppose for get?)***	☐ 1. Yes<br>☐ 2. No<br>☐ 3. Unsure<br><br>☐ 99. Declined to answer	

For the next set of questions (801-811) we would like to learn more about your social support, trusted information sources, and preferred ways of getting information about childhood vaccination services.

(Next question dem we go lek for no bot aw u dae get ep from pipul dem,de sai dem wae u belif for get information,en d way dem wae u dae get information bot u pikin marklate wan)

800	Social support, trusted information sources and preferred communication channels		
ID	QUESTION	RESPONSE	GO TO
801	The last time (NAME) was vaccinated, who took him/her to the vaccination site?	☐1. Mother<br><br>☐2. Father	

	(de last tem wae den marklate d pikin,na wudat bi kerr am go d sie wae den bi marklate tam?)<br><br>(Multiple selection allowed; do not read)	☐3. Grandmother<br>☐4. Grandfather<br>☐5. Sibling<br>☐6. Other relative (specify)<br>☐7. Other non-relative (specify)<br><br>☐ 66. Vaccinated at home during outreach<br>☐ 77. Child has never been vaccinated<br>☐ 88. Don't remember / unsure<br>☐ 99. Declined to answer	
802	Through which means do you <u>usually</u> receive information about childhood vaccination?<br><br>***(wus way u dae get information more bot pikin marklate ?)***<br><br>(Read all, and ask to select one)	☐1. Household visits<br>☐2. Community-based events<br>☐3. Radio programming<br>☐4. Television programming<br>☐5. Social media (e.g. Facebook)<br>☐6. Mosque / church<br>☐7. Health facility (any type)<br>☐8. Pharmacy<br>☐9. Text messages<br>☐10. Voice messaging (on mobile phones)<br>☐11. Other (specify)<br><br>☐ 77. I don't receive information from any of these sources<br>☐ 88. Don't know / unsure<br>☐ 99. Declined to answer	
803	Which of the following means do you <u>prefer</u> the most to receive information about childhood vaccination?	☐1. Household visits<br>☐2. Community-based events<br>☐3. Radio programming<br>☐4. Television programming	

	(pa deh wan ya ,ar dae cam read na ,wus wan u go lek for dae get information bot pikin marklate)<br><br>(Read all, and ask to select one)	☐5. Social media (e.g. Facebook)<br>☐6. Mosque / church<br>☐7. Health facility (any type)<br>☐8. Pharmacy<br>☐9. Text messages<br>☐10. Voice messaging (on mobile phones)<br>☐11. Other (specify)<br><br>☐ 77. I don't have a preference<br>☐ 88. Don't know / unsure<br>☐ 99. Declined to answer	
804	Who do you trust the most to talk to you about childhood vaccination?<br>*(wudat u belief more for tok to bot pikin marklate?)*<br><br>(Select up to three choices)	☐1. Spouse<br>☐2. Child's grandparents<br>☐3. Other relatives<br>☐4. Friends and neighbors<br>☐5. Other parents and caregivers<br>☐6. Community leader (e.g. chief, village leader)<br>☐7. Faith leader<br>☐8. Traditional birth attendant (TBA)<br>☐9. Traditional healer<br>☐10. Community health worker<br>☐11. Health provider (e.g. nurse, doctor, vaccinator)<br>☐12. Pharmacist<br>☐13. Other (specify)<br><br>☐ 77. I don't trust anyone<br>☐ 88. Don't know / unsure<br>☐ 99. Declined to answer	

805	If you could add an additional vaccination site, where would you like it to be?<br><br>*READ ALL ANSWERS. SELECT ONE.*<br><br>*(if u go want for mek dem add one more sai for take maklate, usai u go want mek e dae?)*<br><br>*Near you ose, or near you wok place or near di markit*	☐ 1. Close to home<br>☐ 2. Close to work<br>☐ 3. Close to the market<br>☐ 4. No preference<br><br>☐ 99. Declined to answer	

For the next question, please look at this figure. The biggest circle means "very important", the second circle means "somewhat" important, and no circle means "not at all important". Please point to the circle that relates to how much you feel about every question.

(for d next question, ar wan mek u look den round tin dem ya ,d bigwan mean e important gbain,d second wan mean haf en haf and d last wan mean e nor important at ol,point to d wan wae u think sae e feba u answer)

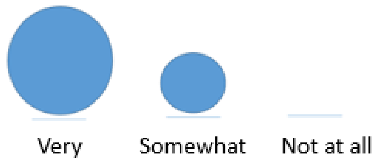

806	How important is it to you that vaccination services are offered on weekends/evenings?<br>*(aw important e be to u for leh den dae gee marklate de weekend or eventem?)*	☐ 1. Very important<br>☐ 2. Somewhat important<br>☐ 3. Not at all important<br>☐ 4. Don't Know<br><br>☐ 99. Declined to answer	

900	***Bednet use***<br><br>*I will now ask you some questions about malaria and malaria prevention…*		
ID	QUESTION	RESPONSE	GO TO
901	Are insecticide-treated mosquito nets provided during routine vaccination visits at your local vaccination site?<br><br>*(De sie wae wuna kin go for marklate wae dae near wuna,den kin gee*	☐1. Yes<br><br>☐2. No<br><br>☐3. Don't Know	

	mosquito net way get meresin wae combra dem kin go for marklate?)	☐99. Declined to answer	
902	Does your household have an insecticide-treated mosquito net?<br><br>(***wuna get tent wae get meresin pa nam na dis hos/pot?)***	☐1. Yes<br><br>☐2. No<br><br>☐99. Declined to answer	2→1001<br><br><br><br>99→1001
902a	How many insecticide-treated mosquito nets does your household have?<br><br>***(armos tent wuna get ?)***	NUMBER OF NETS<br><br>☐☐<br><br>*Enter 88 if don't know*<br><br>*Enter 99 if declined to answer*	
902b	How many insecticide-treated mosquito nets are hanging in your household?<br><br>***(armos pa d tent dem wae u get heng ?)***	NUMBER OF NETS<br><br>☐☐<br><br>*Enter 88 if don't know*<br><br>*Enter 99 if declined to answer*	
903	Where did you get your most recent treated mosquito net from?<br>***(wusai u get d maskita tent wae u get ?)***	☐1. Routine vaccination visit<br><br>☐2. Malaria bednet distribution campaign<br><br>☐3. From a community health worker<br><br>☐4. From a neighbor or friend<br><br>☐5. Purchased one<br><br>☐6. Antenatal care<br><br>☐7. Other<br><br>☐8. Don't know	2,3,4,5→ 906
904	During a routine vaccination visit, do you ever recall receiving mosquito net education on <u>how</u> to use it?	☐1. Yes<br><br>☐2. No	

	(da tem wae den bi gee u da maskita tent den bi tell u how for use am?)	☐3. Don't Know<br>☐99. Declined to answer	
905	During a routine vaccination visit, do you ever recall receiving mosquito net education on why to use it?<br>*(da tem wae den bi gee u da maskita tent den bi tell u why for use am?)*	☐1. Yes<br>☐2. No<br>☐3. Don't Know<br>☐99. Declined to answer	
906	Did (NAME) sleep under a treated mosquito net last night?<br>*((NAME) bin sleep pa maskita tent last net?)*	☐1. Yes<br>☐2. No<br>☐3. Don't Know<br>☐99. Declined to answer	1,3→<br>1001
907	Could you please share why (NAME) did not sleep inside a treated mosquito net last night?<br>*(u able tell mi,why u nor sleep pa maskita tent last net?)*	☐1. Not worried about Malaria<br>☐2. Do not own net<br>☐3. Net used for bathing/fishing/other use<br>☐4. Did not know infant should sleep under a net<br>☐5. Nets are not effective at preventing malaria<br>☐6. Neighbors don't use mosquito nets<br>☐7. It is too hot under the mosquito nets<br>☐8. Nets are used mostly by adults in the household<br>☐9. Other; (SPECIFY)_______________	

1000	DWELLING CHARACTERISTICS		
ID	QUESTION	RESPONSE	GO TO
1001	What is the main source of drinking water for members of your household?	☐1. Piped water<br>☐2. Dug well<br>☐3. Water from spring	

	(wusai na d main sie wae u dae get wata for drink?)	☐4. Surface water (river/dam)<br>☐5. Water tank<br>☐6. Purchase filtered water-------------------------------→	 <br>Q1004
1002	Where is that water source located?<br>*(wusai wuna kin get wata dae?*	☐1. In own home-------------------------------→<br>☐2. In own yard/plot-------------------------------→<br>☐3. Elsewhere	Q1004<br>Q1004
1003	How long does it take to go there, get water, and come back?<br>*(armos for take for go get wata en cam back?)*	☐☐ Minutes<br>☐ Don't know	
1004	What kind of toilet facility do members of your household usually use?<br>*(wus kin toilet wae wuna dae use all tem?)*	☐1. Flush or pour flush toilet<br>☐2. Pit latrine<br>☐3. Bucket/hanging toilet/plastic bags	
1005	What type of fuel does your household mainly use for cooking?<br>*(watin wuna dae use for cook?)*	☐1. Electricity<br>☐2. Log<br>☐3. Natural gas<br>☐4. Biogas<br>☐5. Kerosene<br>☐6. Charcoal<br>☐7. Wood<br>☐8. Straw/shrubs/grass<br>☐9. Agricultural crop<br>☐10. Animal dung<br>☐11 No food cooked	

		☐12. Other	
1006	Does your household have:<br><br>Electricity? ***(wuna ose get light?)***<br><br>A Radio? (***Radio)***<br><br>A television? ***(Television?)***<br><br>A mobile telephone? ***(mobilefone?)***<br><br>A land line telephone? ***(telefone?)***<br><br>A refrigerator? ***(freezer?)***	 <br>☐1. Yes ☐1. No<br><br>☐2. Yes ☐2. No<br><br>☐3. Yes ☐3. No<br><br>☐4. Yes ☐4. No<br><br>☐5. Yes ☐5. No<br><br>☐6. Yes ☐6. No	
1007	Main material of the floor<br><br>ENUMERATOR: OBSERVE (DO NOT ASK)	☐1. Natural floor (ex. earth, sand, dung)<br><br>☐2. Rudimentary floor (ex. bamboo, wood planks)<br><br>☐3. Finished floor (ex. cement, carpet, wood, tiles)	
1008	Main material of the roof<br><br>ENUMERATOR: OBSERVE (DO NOT ASK)	☐1. Natural roofing (ex. thatch, palm, sod)<br><br>☐2. Rudimentary roofing (ex. bamboo, wood planks)<br><br>☐3. Finished roofing (ex. cement, shingles, wood, corrugated iron)	
1009	Main material of exterior walls<br><br>RECORD OBSERVATION	☐1. Natural walls (ex. no walls, cane, palm, dirt)<br><br>☐2. Rudimentary walls (ex. bamboo, mud, plywood, corrugated iron)<br><br>☐3. Finished walls (ex. cement, stone, brick, wood)	
1010	GPS		

Comments: Please add any additional comments or observations about the interview.

END:

Thank you for sharing your experiences and opinions relating to childhood vaccination in your community. The information you provided is valuable, and will help the Sierra Leone Ministry of Health and Sanitation improve childhood vaccination services.

Do you have any questions for me before we end the interview?

- *If YES----------------→Answer question(s)*
    - *if you don't know the answer or feel uncomfortable providing an answer, you should feel free to say so…then ask the respondent to visit the nearest health facility to get more information*
- *If NO---------------→END*
